# Testicular Torsion: Does Age Matter in a 79-Year-Old Patient?

**DOI:** 10.7759/cureus.102402

**Published:** 2026-01-27

**Authors:** Ahmed Anber, Khaled Ghanem, Lewis Mambu

**Affiliations:** 1 Urology, Hereford County Hospital, Wye Valley NHS Trust, Hereford, GBR; 2 Urology, Queens Hospital Romford, London, GBR

**Keywords:** acute scrotum, elderly, scrotal exploration, testicular torsion, us scan

## Abstract

Testicular torsion is a urological emergency typically seen in adolescents, but it can occur at any age, including in elderly men, where it is often misdiagnosed. We present the case of a 79-year-old male who developed acute right testicular pain. Doppler ultrasound confirmed torsion, and timely surgical intervention resulted in testicular salvage. This report emphasises the importance of including testicular torsion in the differential diagnosis for acute scrotal pain, regardless of patient age.

## Introduction

Testicular torsion is an emergency characterised by the twisting of the spermatic cord, leading to compromised blood flow to the testis. It predominantly affects adolescents and young adults, with peak incidence between 13 and 15 years, occurring in approximately 1 in 4,000 males under 25 years of age [1]. In older adults, however, the diagnosis can be easily overlooked, as clinicians may favour more common aetiologies such as epididymitis or orchitis. The consequences of delayed recognition include irreversible ischaemic injury, testicular atrophy, infertility, and hormonal dysfunction [1,2].

Although rare in the elderly, testicular torsion must not be excluded based on age alone. Prompt recognition and surgical intervention are vital to salvage the testis [3-6]. Imaging modalities, particularly Doppler ultrasound, are invaluable tools in this age group due to their non-invasiveness and high diagnostic accuracy, with reported sensitivity and specificity exceeding 93% and 98%, respectively [7]. This report contributes to the limited literature by demonstrating that testicular salvage is achievable even in elderly patients with delayed presentation, reinforcing the need for high clinical suspicion. 

## Case presentation

A 79-year-old male presented to the emergency department with sudden-onset right testicular swelling and pain persisting for three days. The pain was described as a heavy, dragging sensation that progressively worsened and was exacerbated by movement. He denied trauma, dysuria, fever, or prior episodes.

His past medical history included chronic obstructive pulmonary disease (COPD) and prior lung volume reduction surgery. He had no known drug allergies.

On physical examination, the right hemiscrotum was swollen, erythematous, and tender. The testicle appeared elevated and transversely orientated. A surrounding hydrocele was noted. The cremasteric reflex was diminished, and Prehn's sign was negative. No palpable masses were found.

Laboratory investigations revealed an elevated C-reactive protein (CRP) of 40 mg/L and a white blood cell count of 15,000/µL. Urinalysis was negative for nitrites and leukocytes. Serum urea and electrolytes were within normal limits (Table 1).

**Table 1:** Laboratory results Blood test results demonstrating elevated inflammatory markers, which may be misleading in cases of testicular torsion and should be interpreted alongside clinical findings. CRP: C-reactive protein, WBC: White blood cell

Parameter	Patient Value	Reference Range	
CRP	40 mg/L	<5 mg/L	
WBC	15,000 /µL	4,000–11,000 /µL	
Urea and Electrolytes	91 μmol/L	59–104 μmol/L	

**Figure 1:**
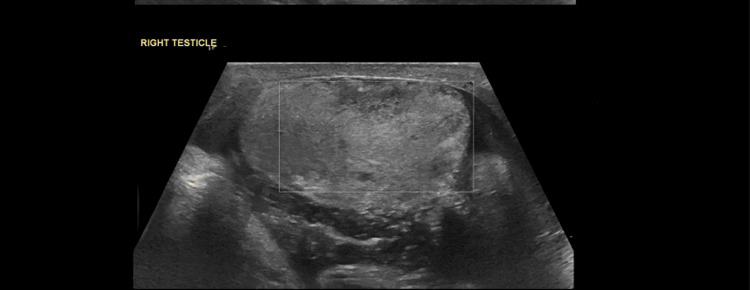
US testis Ultrasound of the right testis showing absent blood supply and early signs of ischemia.

Scrotal Doppler ultrasound demonstrated absent vascularity in the right testis (Figure 1), consistent with testicular torsion.

**Figure 2:**
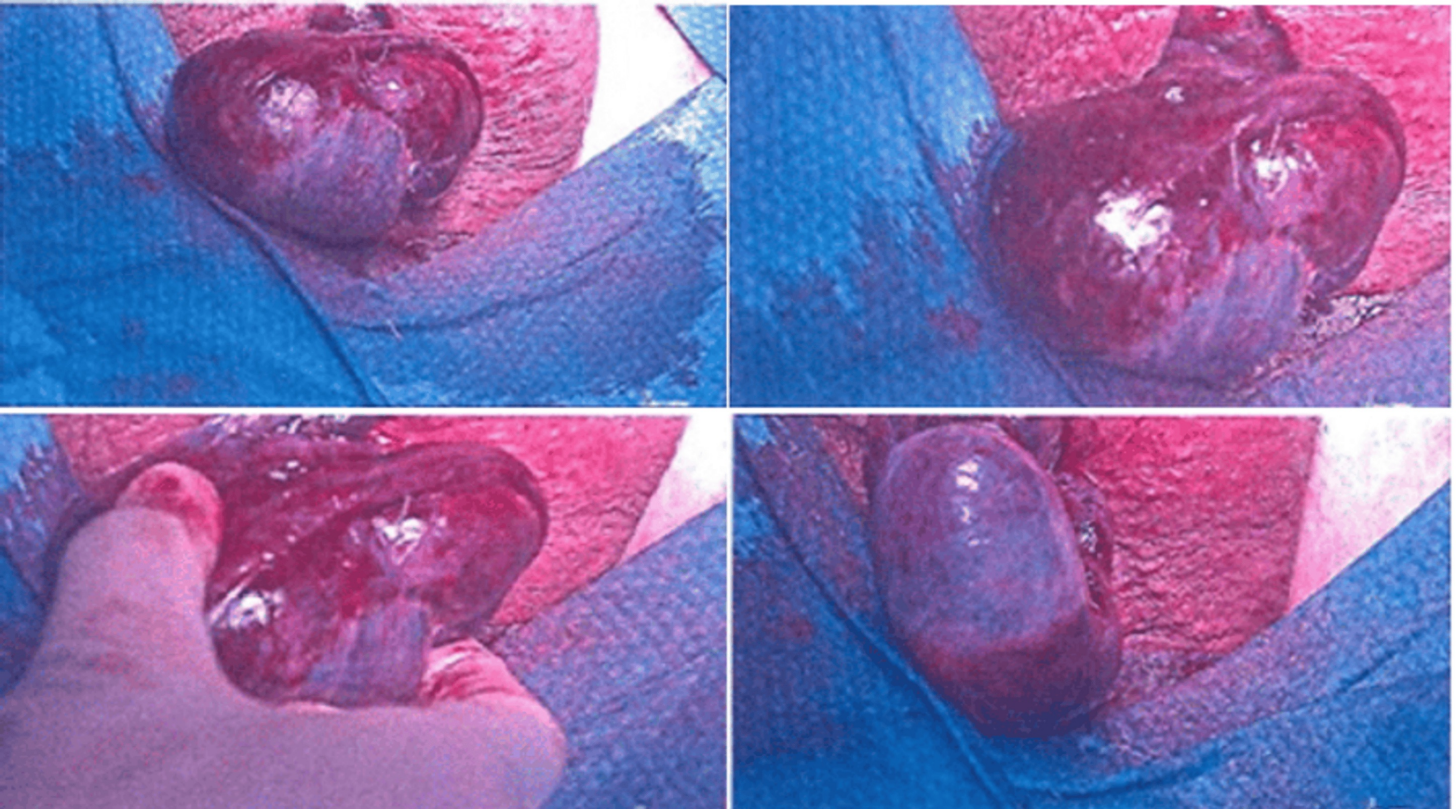
Intraoperative images Intraoperative images of the right twisted testis demonstrating testicular ischaemia

The patient underwent urgent surgical scrotal exploration. Intraoperatively, the right testicle was found to be twisted 270 degrees with a bell clapper deformity. The left testicle was anatomically normal. The affected testis was detorted, and fomentation with warm saline and high-flow oxygen was administered. The tissue colour showed partial reperfusion, and a positive prick test indicated preserved viability based on intraoperative assessment standards. Bilateral orchidopexy was performed using non-absorbable sutures and the three-point fixation technique (Figure 2).

The patient recovered well postoperatively and was discharged with a plan for outpatient Doppler ultrasound of the testes to assess viability and function. However, due to frailty and progression of severe chronic obstructive pulmonary disease, he became housebound and was unable to attend the imaging appointment. Follow-up was therefore conducted via a telephone consultation six months postoperatively, during which he reported no ongoing symptoms or concerns.

## Discussion

Testicular torsion in elderly men remains an under-recognised clinical entity, largely because scrotal pain in this age group is more frequently attributed to infectious, vascular, or neoplastic causes [3,4]. Although congenital anomalies such as the bell clapper deformity are classically associated with adolescents, mounting evidence shows that these anatomical variants may persist into late adulthood and predispose older patients to torsion. Several published reports describe atypical or delayed presentations in elderly men, emphasising that clinical features may be subtle and laboratory markers often misleading, especially when inflammatory parameters are elevated. In many cases, a trial of antibiotics for presumed epididymo-orchitis is initiated, contributing to diagnostic delay and worse salvage rates. The present case aligns with previous observations showing that prompt utilisation of Doppler ultrasound significantly improves diagnostic accuracy, even when physical examination findings are equivocal. Additionally, this case supports emerging literature suggesting that the degree of torsion, duration of symptoms, and intraoperative viability assessments (such as tissue colour and the prick test) are key predictors of salvage success [8]. By detailing the timely diagnosis and favourable outcome in an elderly patient, this report adds to the growing body of evidence that torsion is not exclusively a disease of the young and highlights the need for heightened awareness among clinicians evaluating acute scrotal pain in older adults.

## Conclusions

This case demonstrates that testicular torsion can occur at any age and should not be dismissed solely based on patient demographics. Awareness of its potential occurrence in elderly men is essential, as early recognition and prompt surgical management remain the most important factors for preserving testicular function. The successful outcome in this patient illustrates the value of maintaining a broad differential diagnosis and using Doppler ultrasound early when clinical findings are unclear. By highlighting the possibility of atypical presentations and the persistence of anatomical predispositions into later life, this report reinforces the importance of timely intervention to prevent irreversible ischemic damage and optimise patient outcomes.

## References

[1] Velasquez J, Boniface MP, Mohseni M (2020). Acute Scrotum Pain. https://www.ncbi.nlm.nih.gov/books/NBK470335/.

[2] Schick MA, Sternard BT (2023). Testicular Torsion. https://www.ncbi.nlm.nih.gov/books/NBK448199/.

[3] Mattigk A, Klein JT, Martini T (2020). Testicular torsion in geriatric 82‑year‑old man. Urol Case Rep.

[4] Ali MA, Oyortey M, Maalman RS (2021). Testicular torsion in a catheterized geriatric 73-year-old patient, making an early diagnosis: a case report. J Surg Case Rep.

[5] Allen B, Ball AJ, Desai A (2010). Delayed presentation of acute scrotum: a rare age for torsion. Intern Emerg Med.

[6] Onwuasoanya UE (2021). Atypical presentation of testicular torsion: a case series. Afr Jr Uro.

[7] Ota K, Fukui K, Oba K (2019). The role of ultrasound imaging in adult patients with testicular torsion: a systematic review and meta-analysis. J Med Ultrason (2001).

[8] Rofifa AF, Rifqiawan MZ, Prawira AZ, Shabira NG (2024). Testicular torsion, a time‑challenging urological emergency: a literature review. Wor Jr Adv Res Rev.

